# Pressure–Surface Tension–Temperature Equation of State for *n*-Alkanes

**DOI:** 10.1021/acs.iecr.1c04979

**Published:** 2022-02-23

**Authors:** A. Mulero, I. Cachadiña, L.F. Cardona, J. O. Valderrama

**Affiliations:** † Departamento de Física Aplicada, Universidad de Extremadura, 06006 Badajoz, Spain; ‡ Departamento de Ciencias Básicas, Universidad Católica Luis Amigó, Transversal 51A No. 67B-90, 050031 Medellín, Colombia; § Center for Technological Information (CIT), Monseñor Subercaseaux 667, 1710258 La Serena, Chile

## Abstract

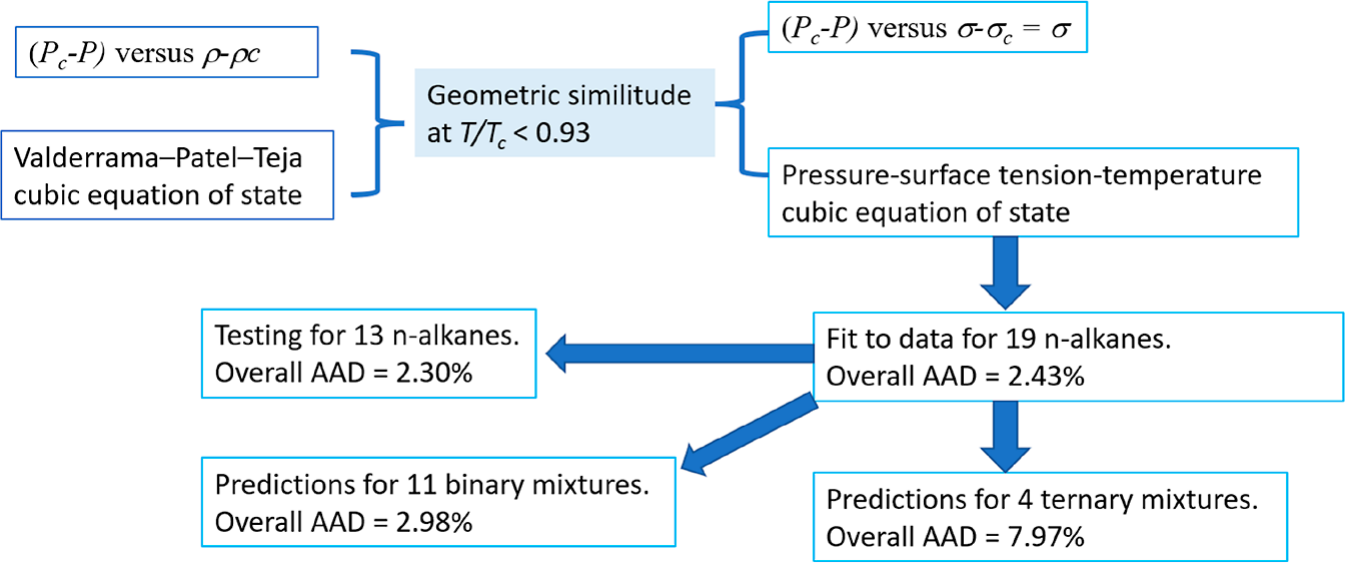

Herein, the geometric similitude concept is applied to propose a cubic equation that relates surface tension, saturation pressure, and temperature for *n*-alkanes. The input properties for each fluid are the molecular mass, pressure, temperature, and compressibility factor at the critical point. The model is applied to temperatures below 0.93·*T*_c_ (critical point temperature). A total of 2429 surface tension values have been selected for 32 *n*-alkanes. The parameters of the model have been obtained with a fit of the surface tension values for 19 pure *n*-alkanes that are randomly chosen. Then, it is tested for the other 13 pure *n*-alkanes and used to predict the surface tension for 11 binary and 4 ternary mixtures. These predictions are compared with the reported experimental data. For pure *n*-alkanes, the overall absolute average deviation is 2.4%, including the correlation and testing sets. No additional adjustable coefficients are used for mixtures, yielding an overall absolute average deviation of 2.98% for the binary systems and 7.97% for the ternary ones. The results show that the model is accurate enough for predictions and that the highest deviations are due to the lack of agreement in the values of surface tension of pure fluids obtained from different sources.

## 1 Introduction

Surface tension is an essential property of liquids, needed for the theoretical and practical studies of different processes such as, for instance, bubble and droplet formation, wetting, capillarity, detergency, atomization, formation of aerosols and sprays, injection of fuels, and so forth.^1−8^

In particular, pure liquid *n*-alkanes and their mixtures are commonly used in some industrial processes, like those including classical combustibles, biofuels, and solvents, which are of high importance and have temperature dependence on their surface tension.^6,9−18^ Thus, it is an essential property; for instance, in the study of the injection of surfactants into the extract crude oil.^6^ In a few words, as the injection process can be affected by the miscibility process, it is needed to estimate the so-called minimum miscibility pressure. This estimation can be made from data or correlations of the interfacial tension of alkanes contained in the crude oil and the injected gases.^1,12,19,20^

Surface tension plays a fundamental role in the injection and atomization of fuels in engines.^9,11,17,21−27^ Thus, *n*-decane is chosen as a surrogate of jet fuels, and its surface tension influences this fuel’s characteristics.^17^ In other cases, liquefied natural gas is used as a fuel, and then the values of the surface tension of alkanes at high pressures are needed to characterize it.^10,28^ Similarly, the knowledge of vapor–liquid equilibrium properties and surface tension of *n*-alkanes and alcohols is also required to study additives commonly used in fuels.^13−15,29,30^

The surface tension of alkanes is also important in removing hydrocarbons from liquid effluents^31,32^ and in the study of carbon dioxide capture and storage technologies.^18,33−35^ In this last case, high pressures and temperatures are needed, which are not always easily accessible. Therefore, it is necessary to use a model with reasonable extrapolation and prediction capabilities.^18,34^

In studying the aforementioned applications, it is necessary to have accurate surface tension data in a wide range of temperatures, especially for the longest or heaviest *n*-alkanes or have reliable and accurate models or methods to calculate or predict them.

The surface tension values can be found in some papers, books, and databases. Still, those data need to be screened and adequately selected, as sometimes there are apparent disagreements between them when taken from different sources. Very recently, Mulero et al.^36^ performed an extensive search to collect the surface tension data available presently for 33 *n*-alkanes, screened them, and finally built a database containing 2561 values. The primary sources used were DIPPR^37^ and DETHERM^38^ databases and Wohlfarth and Wohlfarth’s books.^39−41^ Data from many other books and papers, including some very recently published, were added.^36^

The number of finally selected data for each fluid varied from 9 to 362 and the temperature range covered ranged from 15 to 99% of the whole vapor–liquid equilibrium range (the critical point was not considered, as the surface tension was zero at that point). Finally, they proposed specific correlations (containing two or four adjustable coefficients for each fluid) that reproduce the selected data with mean absolute percentage deviations for each fluid below 2.1% and percentage deviations for each data below 10% except for 9 of them (which are close the critical point).^36^ The correlation models have not been applied to model or predict results for mixtures.

Apart from these specific correlations, it would be desirable to have general models with coefficients valid for at least a family of fluids, with predictive capacity, and applicable to mixtures. Some semitheoretical methods, such as computer simulations, the application of the gradient theory together with an equation of state (EoS), and others have been already applied to the surface tension of *n*-alkanes and some mixtures containing them.^14,15,32,34,42−55^ Their main advantages are that they are based on some theoretical approaches and that, apart from the surface tension, other interface properties can be calculated. However, as expected, these general models are sometimes not accurate enough. Their application requires specific software or the development of complex computer programs,^55^ so they cannot be considered straightforward ones.

A comprehensive summary of these models has been recently performed in ref (18). In particular, the gradient theory together with an EoS is one of the most used.^14,15,32,34,42,45,49−53,55^

Two examples of the use of this kind of model for pure fluids can be mentioned here. One is the molecular parametrization based on a new version of the statistical associating fluid theory (SAFT) EoS proposed by Mejía et al.^51^ Qualitative comparison of the results obtained for the surface tension of *n*-hexane and 5-nonanone showed a good agreement when compared with some experimental results. Following a similar procedure but using a different version of the SAFT EoS, Garrido et al.^52^ obtained results for the surface tension of 15 pure fluids, including some *n*-alkanes. The deviations with respect to experimental results were below 4.8%, the overall absolute average deviation being 2.4%. As is said, a summary of the results obtained with this kind of model for different kinds of fluids is available in ref (18).

Another alternative is to consider purely empirical or semiempirical methods such as artificial neural networks, group-contribution methods, quantitative structure–property relationships, corresponding states’ principle (CSP) methods, or their combinations. These methods have also been applied to the surface tension of *n*-alkanes.^54,56−78^ Nevertheless, the results are not satisfactory because of the limited number of fluids, data, or both. In many cases, a reduced number of data sources (or even just one) were studied, and in many cases no previous selection or comparison between them was made.

This paper proposes a generalized model with adjustable coefficients fixed for all the considered *n*-alkanes. Previously, in the following paragraphs, as well as in Table S1 (pure fluids and including *n*-alkanes) and Table S2 (*n*-alkanes mixtures) of the Supporting Information, we summarized the results obtained by other authors that have used generalized pure empirical or semiempirical models for the calculation of the surface tension of organic substances.

In 1995, Sastri and Rao^56^ proposed a corresponding state correlation for the surface tension of pure liquids, including three general adjustable coefficients. The model was applied to some *n*-alkanes and other substances, but the temperature ranges studied were narrow, and only one data source was considered. As can be seen in Table S1, the obtained mean percentage deviations were not low, so the model has not been subsequently applied.

In 1997, Zuo and Stenby^57^ proposed a new CSP model that utilized the surface tension of methane and octane as reference. It was applied to 86 fluids of different kinds. The data selected for each *n*-alkane ranged from 7 to 42, the temperature ranges considered were not very wide, and the most updated data were from 1992. As shown in Table S1, the obtained absolute average deviations (AADs) for *n*-alkanes from ethane to eicosane were in the range of 0.5–11.4%.

In 2000, Miqueu et al.^59^ made a literature survey of the published experimental data for the surface tensions of *n*-alkanes from methane to *n*-octane and nitrogen and *i*-butane. They observed apparent differences between the data offered from different sources and selected the most suitable one for some of these fluids. They proposed a new CSP expression, which gave an overall AAD of 3.7%.

From 2001 to 2005, Queimada et al.^61−64^ studied the surface tension of *n*-alkanes and their binary mixtures, performing new measurements and proposing some CSP models (see Table S1). The first model^61^ was similar to that of Zuo and Stenby^57^ but used three fluids as a reference: hexane, undecane, and pentadecane. The MAPDs for 19 *n*-alkanes (from ethane to hexacontane) were from 0.21 to 5.48%, and the mean value was 1.14% (see Table S1). The second model was applied to 18 *n*-alkanes and gave an overall MAPD of 3.7%.

Gharagheizi et al.^69^ proposed two corresponding state models for the surface tension of all the fluids included in the DIPPR database (about 1700 compounds). Not all the fluids or data were included in the fitting procedure, and the overall MAPDs were excessively high (18 and 25%, respectively). For the *n*-alkane family, the overall AADs were 4.9 and 2.7%, respectively. The main drawback of these results is that only the DIPPR data were considered, without any comparison with other sources.

The previously mentioned models are based on the CSP. As shown in Table S1, other kinds of empirical models have also been proposed for *n*-alkanes. Nevertheless, in most cases, the number of substances or data considered is not high enough. In those models in which more than 20 *n*-alkanes were considered, the obtained overall AADs were high (this is the case of Aleem et al.^72^ and Aleem and Mellon^76^ models), or the number of adjustable parameters was very high (ANN model by Lashkarbolooki and Bayat^77^).

By checking the results obtained with the models, as listed in Table S1, one can see that the overall AADs are generally below 6%, being around 2–4% in most cases, with AAD values for each fluid below 10%. On the other hand, it is necessary to consider that, in most models, the data sources were not updated enough, or the data came from just one source (especially for the papers published after 2001).

The main empirical or semiempirical models applied to mixtures of *n*-alkanes and their results are summarized in Table S2.^57,62−64,79,80^ In this case, the number of models is low compared to those for pure fluids. As can be seen, Zuo and Stenby’s model^57^ was extended to mixtures obtaining good general predictions without using adjustable coefficients. Five binary and two ternary mixtures were composed only for *n*-alkanes, for which AADs ranged from 0.77 to 3.21%.

In 2002, Rolo et al.^79^ made experimental surface tension measurements for four binary mixtures of *n*-alkanes. They applied a corresponding state model with three reference fluids (using two adjustable coefficients for each one) and then predicted their experimental data with an overall AAD below 1%. This success was partly because the reference fluids were the same as those present in the mixtures.

In 2003, Queimada et al.^62^ made new measurements for the surface tension of heptane, eicosane, docosane, tetracosane, and some of their mixtures. The model proposed for pure fluids was applied to the binary mixtures, finding a good agreement (see Table S2) using just one adjustable exponent valid for all the mixtures. In a subsequent paper, Queimada et al.^64^ performed new measurements for the surface tension of decane, eicosane, docosane, and tetracosane; their three binary mixtures; and a ternary mixture. Using the same model developed previously,^62^ without any new adjustable parameter, the obtained overall AAD was 1.2% and the maximum MAPD was 2%.

Finally, in 2013, Ghasemian^80^ applied the Sprow–Prausnitz equation to predict (without using adjustable parameters) or reproduce (using two adjustable parameters) the surface tension of 154 binary mixtures at one selected temperature. The results are summarized in Table S2. For the five mixtures of *n*-alkanes, two adjustable parameters were used. The obtained AADs were very low, an expected result, as only five data were considered for each mixture.

Apart from these empirical models, some semitheoretical models have been applied for mixtures containing *n*-alkanes. In particular, the combination of the square gradient theory, the SAFT EoS, and molecular dynamics results were used by Müller and Mejía^45^ for three asymmetric binary mixtures composed of long *n*-alkanes in equilibria with a smaller solvent: hexane + decane, carbon dioxide + decane, and ethane + eicosane. Pure component data were used to fit model parameters, whereas the results for mixtures were predictions. The results differ for each mixture. Thus, the AAD was 1.10% for hexane + decane but 13% for carbon dioxide + decane when compared with experimental results. In the case of ethane + eicosane, the comparison was with computer simulation results, the AAD being 5.56%.

Moreover, Fu et al.^81^ used the perturbed-chain version of SAFT EoS to describe the phase behavior of binary methane–*n*-alkane mixtures. The surface tension of the binary systems methane–propane, methane–pentane, methane–heptane, and methane–decane was satisfactorily predicted, but the comparison made was only qualitative (percentage deviations were not calculated).

Cumicheo et al.^82^ obtained experimental results for mixtures of carbon dioxide with dodecane, tridecane, and tetradecane at 344.15 K and proposed a model based on the use of another version of the SAFT EoS. For the two first mixtures, the overall AAD given for the model with respect to the experimental results was 7.5 and 8.5%, respectively. For the third mixture, the value reduced to 3.3%.

Garrido et al.^53^ used the SAFT EoS and molecular dynamics simulations to model four nitrogen + *n*-alkane mixtures (from pentane to octane). The surface tension was predicted with AAD below 1.5% for the two first mixtures and below 5% for the fourth when compared with experimental results (no experimental results were available for the third). Deviations around 3% were obtained when the model was compared with results from computer simulations.

Subsequently, Garrido and Polishuk^83^ have used the critical-point-based perturbed-chain SAFT equation by implementing standardized and transparent parametrization procedures to obtain the surface tension of water, some *n*-alkanes, carbon dioxide, and nitrogen. A qualitative comparison with the experimental results was made for the surface tension of six mixtures of carbon dioxide with *n*-alkanes, obtaining acceptable results in most cases.

In sum, it is clear that different kinds of general models are presently available for the surface tension of *n*-alkanes. Nevertheless, in most cases, the models were developed using only one source of data or a reduced number of them, a narrow temperature range, or a limited number of *n*-alkanes. Moreover, new data have been published for *n*-alkanes during the last few years, so it is convenient to consider new models with suitable characteristics such as (i) based on an updated and adequate selection of data; (ii) applicable in a wide range of temperatures; (iii) take into account that at high temperatures, the surface tension has to be measured at pressures higher than the atmospheric one; (iv) can be used for predictions; and (v) can be easily extended to mixtures providing good overall results.

An approach that several authors have explored is the use of well-known equations of state. The pressure–volume–temperature relationship is replaced for a pressure–new property–temperature one. This procedure, known as geometric similitude, is based on the two-dimensional similarity between the diagrams of the old and new properties. Thus, new pressure–viscosity–temperature and pressure–thermal conductivity–temperature cubic EoS’s have been recently proposed.^84−87^ In these equations, the density is replaced for the new property to be calculated.

More recently, Cardona and Valderrama^88^ have applied the same idea to the surface tension of ionic liquids. They observed the geometric similitude between the density–temperature and surface tension–temperature diagrams in some temperature ranges, in which both properties are practically linear with the temperature. They used the Valderrama–Patel–Teja (VPT) cubic EoS,^89^ where surface tension replaces density with a suitable redefinition of the required constants, coefficients, and input properties. One of the advantages of using this kind of EoS is that, once the temperature and pressure are fixed, their roots (the surface tension in this case) are obtained analytically by using the so-called Cardano’s expression.^90^ No numerical methods are needed.

The new pressure–surface tension–temperature relationship proposed by Cardona and Valderrama^88^ was applied to model the surface tension of pure ionic liquids and successfully extended to binary and ternary mixtures. In all cases, the data used were those available at atmospheric pressure, so values at temperatures higher than the normal boiling point were not considered.

This paper aims to establish a new geometric similitude for the surface tension of *n*-alkanes in which the data above the boiling point could be included. Thus, a new pressure–surface tension–temperature relationship is proposed, based on the VPT cubic EoS. The adjustable coefficients are obtained for a set of *n*-alkanes and then tested by comparing the obtained values with the data selected for other *n*-alkanes. Finally, predictions are made for some binary and ternary mixtures. The requirements previously mentioned as (i) to (v) are fulfilled with this procedure.

## 2 Cubic Equation for Pressure–Surface Tension–Temperature

As stated by Cardona and Valderrama,^88^ the geometric similitude concept can be more difficult to visualize in the surface tension than for other properties such as viscosity or thermal conductivity.

They found a geometric similitude between surface tension and liquid density in the case of ionic liquids in narrow temperature ranges, where both properties are practically linear with temperature. Nevertheless, this similitude cannot be extended to higher temperatures, where the density tends to its critical point value. In contrast, the surface tension tends to zero (its value at the critical point).

It is necessary to consider that, at low temperatures (below the boiling point), the pressure at which the surface tension is measured or calculated is just the atmospheric one. This pressure must be higher than the atmospheric one and be precisely the liquid–vapor saturation pressure for temperatures higher than the boiling point.

By considering the two physical aspects mentioned above, the geometric similitude proposed here is established between the difference between the liquid saturation density and its value at the critical point (their maximum value indeed), (ρ–ρ_c_), and the surface tension (as its value at the critical point is zero), σ–σ_c_ = σ.

**Figure 1:**
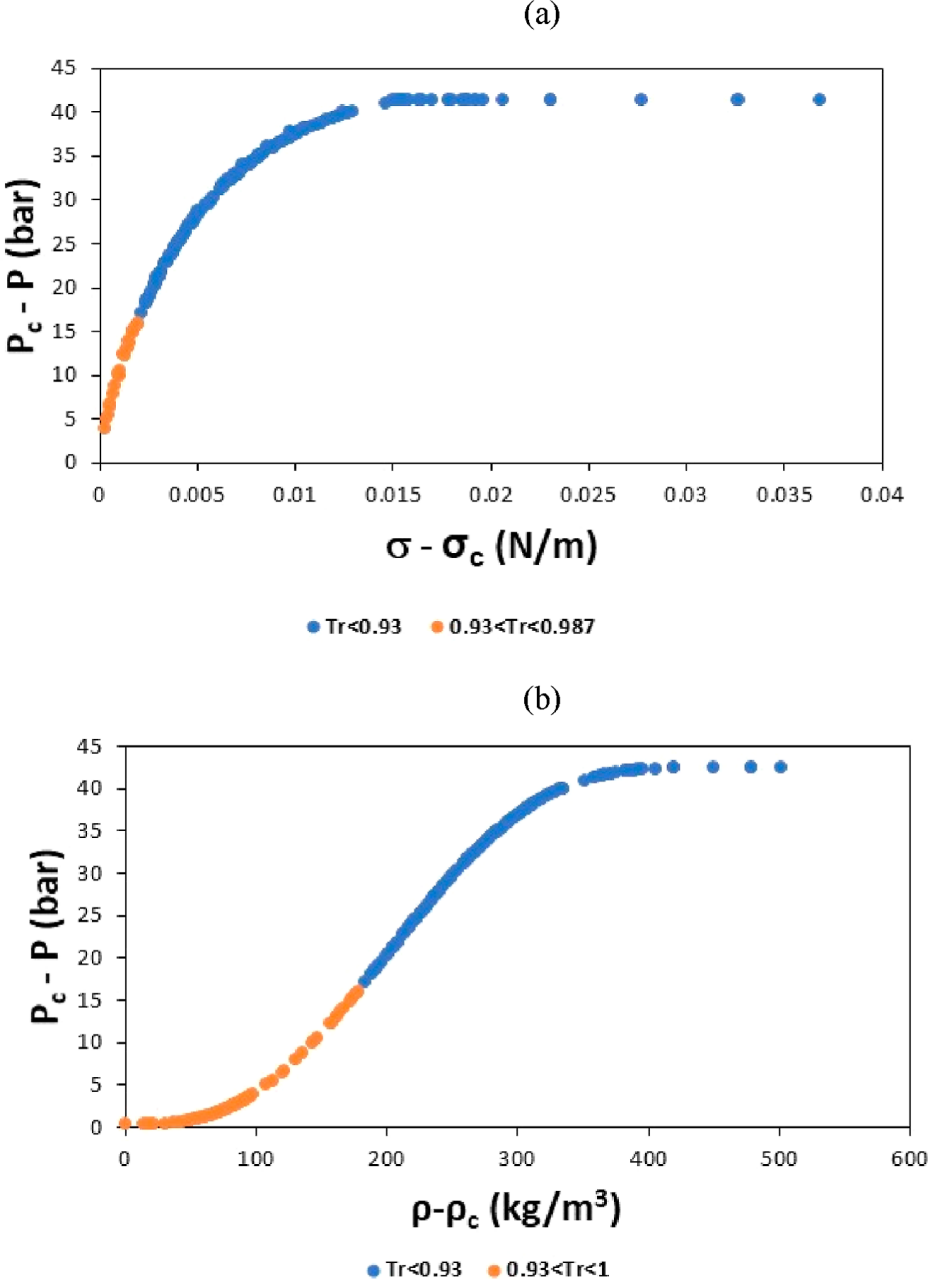
Comparison of the thermodynamic diagrams for propane: (a) (*P*_c_–*P*) vs ρ–ρ_c_; (b) (*P*_c_–*P*) vs surface tension, σ–σ_c_ = σ. Data for saturation pressures and densities were taken from the DIPPR^37^ correlation. Data for the surface tension were taken from the compilation made by Mulero et al.^36^ The data at reduced temperatures (*T*/*T*_c_) higher than 0.93 (those in orange) were not considered in the model.

Figure 1 shows an example of this similitude for propane, where the diagrams of (*P*_c_–*P*) versus (ρ–ρ_c_) and versus the surface tension, σ, are represented. In the case of the (*P*_c_–*P*) versus (ρ–ρ_c_), the saturation pressures and densities were obtained using the temperature correlations from DIPPR^37^ at the corresponding surface tension temperatures selected by Mulero et al.^36^ In the case of *P*_c_–*P* versus σ–σ_c_ = σ, it is necessary to consider that the surface tension is measured below the boiling point temperature at atmospheric pressure. Thus, in Figure 1b, (*P*_c_–*P*) is a constant for temperatures below the normal boiling point and the difference between the critical pressure and saturation pressure for higher temperatures. In Figure 1, the *P*_c_ value is taken from DIPPR.^37^

As can be seen, the geometrical similitude is better appreciated far from the critical point, that is, at low temperatures, where (*P*_c_–*P*) is almost constant in Figure 1a and constant in Figure 1b. At intermediate temperatures, the curvatures are similar, but near the critical point [high temperatures and (*P*_c_–*P*) going to zero], the density and surface tension data trends are different. In the example shown in Figure 1, the differences could be appreciated only at values of (*P*_c_–*P*) below approximately 15 bar. In the case of propane, shown in the example, this zone corresponds to temperatures higher than approximately 0.93 times the critical point temperature, *T*_c_. Because of this, the application of the here-proposed model will be limited to the range *T*_r_ = *T*/*T*_c_ < 0.93.

It must be taken into account that the surface tension is almost zero at higher temperatures, so any tiny deviation between the calculated value and the available data in experiments will lead to a significant percentage deviation. In other words, any model will produce high percentage deviations in calculating the surface tension at temperatures very near the critical point.^36,59,61−64^

In sum, the following hypothesis is established here: an EoS that can well represent the effects of pressure–density–temperature relationship will also be able to describe the effect of pressure and temperature on the surface tension, using the concept of geometric similitude. This hypothesis was already demonstrated by Cardona and Valderrama^88^ for pure ionic liquids and binary and ternary mixtures for temperatures below the boiling point. Here, the assumption is extended to higher temperatures by considering the variable (*P*–*P*_c_) instead of directly *P* in the EoS. The EoS considered is the VPT one, written in the analytical form is given in Table 1. In this new expression, the meaning of the parameters is entirely different from that in the original VPT EoS. Therefore, it has no sense to seek any relation between the order of magnitude, behavior, units, sign, and so forth, of the original and the new parameters defined here.

**Table 1:** Proposed Pressure–Surface Tension–Temperature Model Based on the VPT EoS

description	mathematical expressions
explicit pressure expression	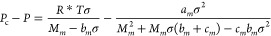 A1
EoS parameters	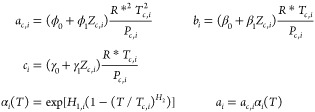 A2
fitted parameters	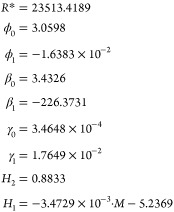 A3
simple van der Waals mixing rule	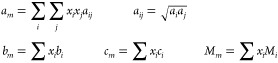 A4

In eqs A1 and A2 of Table 1, *P* is the pressure in bar, *T* is the temperature in kelvin, σ is the surface tension in newton per meter, *M* is the molar mass (g/mol), and *Z*_*c*_ is the critical compressibility factor (dimensionless). The function α_*i*_(*T*) is that proposed by Heyen,^91^ being *H*_1*i*_ fluid dependent, and *H*_2_ fitted considering the whole set of substances. The parameters ϕ_0_, ϕ_1_, β_0_, β_1_, γ_0_, γ_1_, and R* are fixed coefficients with values obtained for the selected *n*-alkanes.

It is essential to clarify that although in the original VPT EoS, where the parameters may have a certain physical meaning, the model parameters do not necessarily have a physicochemical sense in the extended model based on the geometric similitude concept.

The mole fraction in mixtures is represented by *x*_*i*_. The subscripts *m*, *c*, *i*, and *j* indicate the mixture, the critical properties, and the components “*i*” and “*j*” respectively. The simple mixing rules in Table 1 are the same as those used previously by Cardona and Valderrama^88^ for mixtures of ionic liquids. No adjustable coefficients are introduced in them, so the new equation is used as a predictive tool for mixtures.

The surface tension is obtained for a fixed *T* and *P* by solving eq A1 in Table 1. For that, a cubic polynomial, with coefficients *f*_0_, *f*_1_, and *f*_2_, is constructed (see Table 2). Then, the parameters *p* and *q* of Cardano’s method^90^ are calculated. The cubic equation provides three real roots, and because the obtained property (surface tension) is defined for the liquid phase, the highest root is always selected as the proper solution.^88^ No numerical procedures are needed.

**Table 2:** Solution of the Empirical Model Using Cardano’s Analytical Method

mathematical expressions of the general model	coefficients of the cubic polynomial equation
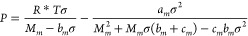	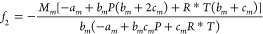
cubic polynomial	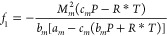
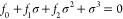	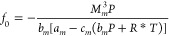
Cardano’s analytical solution	associated parameters of Cardano’s method
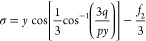	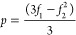
	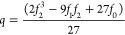

## 3 Data Selection and Calculation of Parameters

The database used here is practically the same as selected recently by Mulero et al.,^36^ which is based on values obtained from databases, books, and a high number of papers. In particular, only 32 *n*-alkanes are considered here out of the 33 studied by Mulero et al. Tetracontane has not been included here because all the required constants properties for the application of the EoS model (*M*, *T*_c_, *P*_c_, and *Z*_c_) are taken from DIPPR database, and this liquid is not in this database.

The available data for each fluid were carefully checked and selected. When the same data set is found in different sources, it is taken into account only once. If a datum or data set were in an apparent disagreement with most of the remaining data, they were excluded. In some cases, there are different trends for the data coming from different sources. If possible, the data that follow approximately the same trend were selected, whereas others were discarded. All the details are explained in ref (36).

Apart from discarding the data for tetracontane, the application of the model was limited to the reduced temperatures below 0.93, as explained in the previous section. This restriction affects only the lighter *n*-alkanes, from ethane to octane, for which data at higher temperatures are available.

Thus, the set of fluids considered here includes 32 *n*-alkanes for which 2429 data were selected (details of selection and origin of data can be seen in ref (36)). The data selected for each fluid change drastically from one to another liquid, ranging from 9 to 339. It must be noted that the data are not entirely homogeneous for most fluids, as they come from different sources. This means that two or more other values for the surface tension can be included at the same temperature. The disagreement between these values is not significant in general but can lead to relatively high percentage deviations compared with the value provided by a model.

Values of the constant properties for each *n*-alkane are given in Table S3 as the Supporting Information. The number of surface tension data, pressure, and temperature ranges of selected data, and surface tension ranges are shown in Table 3. This last table includes the normal boiling point temperature, *T*_b_, (as given in DIPPR^37^), as the model requires the saturation pressure for temperatures higher than it. It must be noted that those fluids with surface tension data are available only at the atmospheric pressure have a maximum temperature range below *T*_b_.

**Table 3:** Number of Data, Ranges of Pressure and Temperature, and Percentage Deviations^a^

	substance	*N*	*T*_b_ (K)	Δ*P* (bar)	Δ*T*_r_	Δ*T* (K)	Δσ (mN/m)	AAD (%)	MAXD (%) [*T*m (K)]
correlation	methane	117	111.66	1.01–29.17	0.48–0.93	90.95–176.41	1.553–16.894	2.26	7.75 [174.19]
	ethane	141	184.55	1.01–30.73	0.31–0.93	93.15–283.92	1.85–32.9	4.66	10.65 [259.38]
	butane	109	272.65	1.01–22.17	0.32–0.92	136.19–393.15	2.091–34.21	2.13	9.98 [393.15]
	pentane	137	309.22	1.01–18.91	0.31–0.92	144.18–433.15	2.24–33.76	1.58	9.95 [432.4]
	nonane	78	423.97	1.01–1.12	0.38–0.72	223–427.79	11–29.57	0.86	4.07 [293.15]
	decane	149	447.305	1.01–11.32	0.4–0.93	248–573.15	1.94–27.96	1.27	14.87 [573.15]
	undecane	60	469.078	1.01–3.25	0.43–0.82	273.15–523.15	5.85–26.58	0.83	7.92 [523.15]
	tridecane	48	508.616	1.01	0.4–0.66	273.15–443.15	13.71–27.87	1.78	4.09 [443.15]
	tetradecane	49	526.727	1.01	0.41–0.76	283.15–526.59	9.33–27.6	2.31	9.11 [526.59]
	pentadecane	40	543.835	1.01	0.4–0.6	283.15–423.15	16.9–28.1	2.58	5.98 [423.15]
	eicosane	25	616.93	1.01–1.03	0.41–0.8	313.15–618.12	7.92–27.62	2.27	15.15 [618.12]
	heneicosane	22	629.65	1.01–1.71	0.4–0.84	313.35–657.22	5.013–27.314	3.62	9.14 [553.15]
	docosane	28	641.75	1.01–1.57	0.4–0.84	317.15–664.84	5.091–27.817	3.89	9.22 [553.15]
	tetracosane	31	664.45	1.01–1.36	0.4–0.84	323.75–679.17	4.933–27.05	2.59	8.08 [573.15]
	hexacosane	28	685.35	1.01–1.16	0.4–0.84	329.25–691.78	4.5973–27.701	1.53	13.24 [691.78]
	heptacosane	10	695.25	1.01–1.17	0.4–0.85	332.15–702.27	4.716–26.928	2.51	7.55 [702.27]
	octacosane	17	704.75	1.01	0.4–0.84	334.35–702.75	4.862–26.639	3.97	8.86 [448.15]
	triacontane	15	722.85	1.01	0.4–0.84	338.65–712.83	4.9385–27.091	3.87	8.93 [423.15]
	dotriacontane	12	738.85	1.01	0.41–0.49	346.35–422.45	21.7–27.2	1.69	2.74 [412.45]
testing	propane	170	231.11	1.01–25.31	0.26–0.92	95–342.03	2.172–36.81	2.67	9.26 [95]
	hexane	259	341.88	1.01–15.26	0.35–0.91	178–463.28	2.43–31.42	1.53	15.28 [463.15]
	heptane	339	371.58	1.01–15.24	0.34–0.93	183–500	2.1423–32.28	1.27	12.59 [497.06]
	octane	194	398.83	1.01–12.91	0.38–0.92	218–523.15	2.32–29.26	0.96	6.87 [523.15]
	dodecane	100	489.473	1.01–5.3	0.42–0.87	273.15–573.15	4.14–27.24	0.96	6.66 [298.16]
	hexadecane	118	560.014	1.01–1.34	0.41–0.79	293–573.15	7–28.12	2.78	6.49 [373.15]
	heptadecane	34	575.3	1.01	0.41–0.64	298.15–473.15	14.24–27.64	1.76	4.91 [473.15]
	octadecane	29	589.86	1.01	0.41–0.59	303.15–443.15	16.58–27.59	1.75	3.68 [305.15]
	nonadecane	12	603.05	1.01–1.04	0.41–0.8	313.15–604.55	7.6–26.91	0.95	2.28 [313.15]
	tricosane	30	653.35	1.01–1.48	0.4–0.84	320.65–672.43	4.965–27.169	2.68	8.43 [573.15]
	pentacosane	9	675.05	1.01–1.25	0.4–0.84	326.65–685.89	4.964–27.125	2.00	5.77 [685.89]
	nonacosane	10	713.95	1.01	0.4–0.85	336.85–712.46	4.7–26.778	3.21	8.8 [712.46]
	hexatriacontane	9	770.15	1.01	0.4–0.84	349.05–737.98	4.8216–26.348	7.37	11.27 [446.28]

a *T*_b_ is the value of the normal boiling point temperature reported by DIPPR.^37^ Atmospheric pressure is written as 1.01 bar. AAD (%) is defined in eq 1. MAXD is the maximum value of PD_*i*_ and [*T*_m_ (K)] is the temperature at which this maximum is reached. The substances are sorted by number of carbons.

The 32 *n*-alkanes were randomly divided into two subsets to evaluate the model’s accuracy. Although the percentage of data used for correlation and testing is different from that used by other researchers;^85−88^ here, the results of the testing process for the *n*-alkanes were privileged. Thus, approximately 60% of the fluids were used in correlation and 40% in the testing process. In particular, a subset of 19 substances was used to obtain the adjustable parameters, whereas the data for the other 13 *n*-alkanes were used to test the model’s accuracy. The fluids assigned to each subset were chosen randomly. In Tables S3 and 5, the fluids are sorted by the number of carbons into two separate lists, one for the correlation process and the other for testing.

The parameters defining the model are ϕ_0_, ϕ_1_, β_0_, β_1_, γ_0_, γ_1_, R*, and *H*_2_, which are considered as constants valid for all the selected fluids. *H*_1_ takes a different value for each fluid. To obtain them, the 1116 surface tension data compiled by Mulero et al.^36^ at reduced temperatures below 0.93 were selected for the first 19 *n*-alkanes listed in Table 3.

The generalized reduced gradient optimization method is used to find the optimum values of the model constants incorporated in Solver of MS Excel.^92^ This method converges to acceptable accurate solutions according to the results presented in the literature.^92−94^ The objective function to be minimized is the mean of the average absolute relative deviations (AADs) between the values determined by the EoS model and the selected data for each fluid (i.e., the overall AAD defined as the sum of AADs for the selected fluids divided by the number of fluids). The AAD is obtained for each fluid as follows
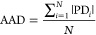
1where PD_*i*_ is the percentage deviation for each data
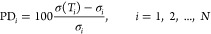
2σ(*T*_*i*_) being obtained from the model and σ_*i*_ being the selected value at the same temperature, *N* is the number of data for each fluid.

**Figure 2:**
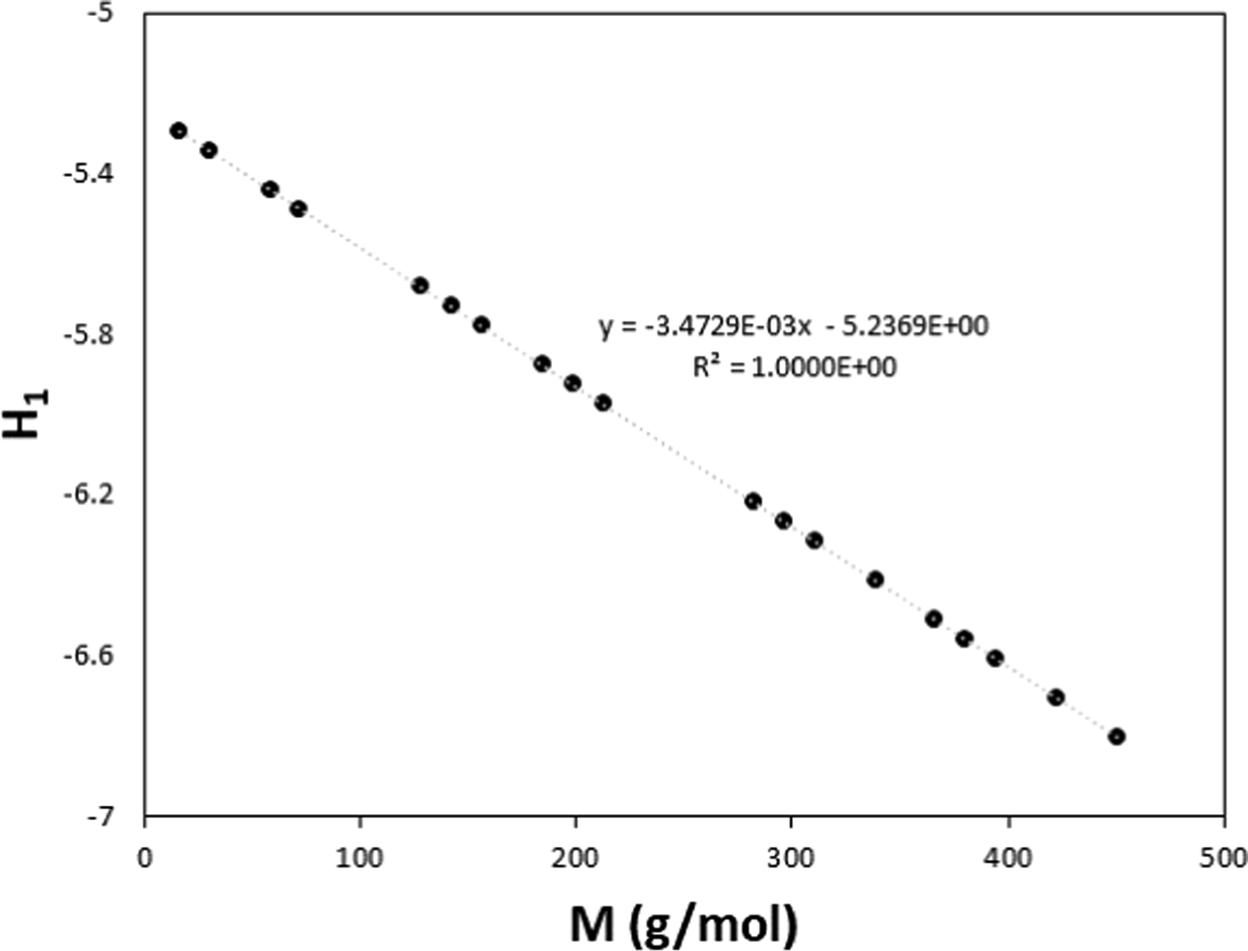
Values of *H*_1_ for the *n*-alkanes included in the correlation set vs their molar mass. The analytical expression and the correlation coefficient are given.

The adjustable parameters were firstly obtained using this procedure. The values for H_1_ for the 19 first fluids in Table 3 (correlation set) are shown in Figure 2 as a function of the molar mass (*M*). As can be seen, this parameter can be obtained very accurately as a linear function of *M*. Then, two new constant parameters are introduced for the analytical expression of *H*_1_ as a function of *M*. Finally, the constant parameters were calculated again by including the new two adjustable parameters to obtain *H*_1_. The final calculated values are those shown in eq A3 of Table 1.

Therefore, finally, the model requires the previously considered constant parameters, the *M*, *T*_c_, *P*_c_, and *Z*_c_ values for each fluid, and the saturation pressure when the temperatures are higher than that of the boiling point. The surface tension values are obtained analytically by using Cardano’s method,^90^ as given in Table 2.

In this paper, the model has been applied first to the 19 *n*-alkanes considered in the correlation set and then to the other 13 *n*-alkanes in the testing set. Some results have also been obtained at reduced temperatures higher than 0.93, not considered previously in the definition of the model. Finally, predictive results were obtained for 11 binary mixtures and 2 ternary mixtures.

## 4 Results and Discussion

As explained in the previous section, the overall AAD for the correlation set was the objective function. The results obtained for each pure fluid are given in Table 3. Apart from the AAD for each fluid, the maximum percentage deviation (MAXD) for a datum (i.e., the maximum value of |PD_*i*_|), and the temperature at which this maximum occurs, *T*_m_, are also shown.

The results for the pure fluids used in the correlation process are considered here first. Then, the ones for testing set are shown and discussed. Later, the predictions for some high-temperature data excluded in the model’s definition are considered. Finally, predictive results for binary and ternary mixtures are shown and analyzed.

### 4.1 Pure *n*-Alkane Results for the Correlation Set

As shown in Table 3, for the 19 *n*-alkanes used to obtain the model, the number of data considered goes from 12 to 149. The obtained AADs range from 0.83 to 4.66% and the overall value is 2.43%. For 14 of these fluids, the AAD is below 2.6%, which can be considered an excellent result. On the other hand, MAXD values are below 10% except for 4 fluids, and the highest data deviation is 15.15%.

The highest AAD value for the correlation set (4.66%, the only value higher than 4% for these fluids) corresponds to ethane, which is the second fluid with the highest number of data (141) located at the highest temperature range (*T*_r_ = 0.31–0.93) selected. The ARD has practically the same value, which means that the model overpredicts the selected data in all the temperature range. Mulero et al.^36^ have proposed specific correlations for the *n*-alkanes, being the highest AAD also obtained for ethane. As they commented, the wide temperature range covered makes it difficult to reproduce the data at low and high temperatures with enough accuracy. In any case, using the here-proposed model (defined in Table 1), only 2 out of the 141 data selected have PD_*i*_ values higher than 10%. An AAD value below 5% can be considered acceptable when considering the number of data and temperature range covered.

**Figure 3:**
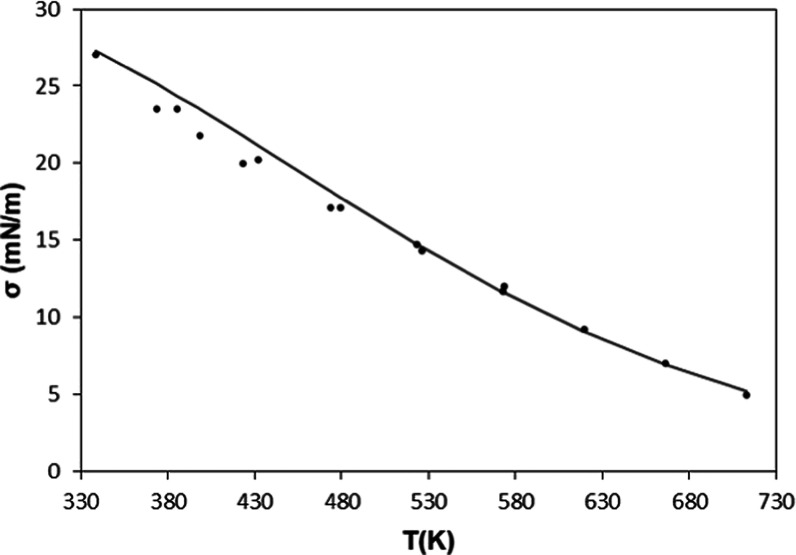
Surface tension values for triacontane versus temperature. Points: selected data. Line: results for the model.

**Figure 4:**
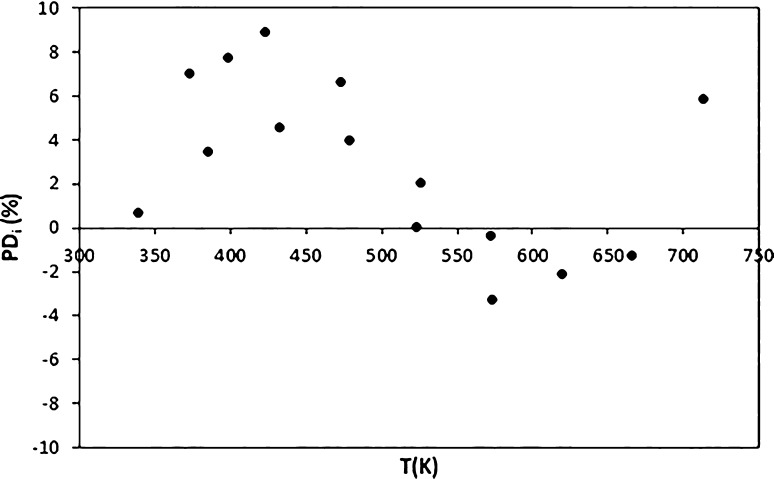
Percentage deviations between the values for the surface tension of triacontane obtained with the model and those selected in the database.

There are three fluids in the correlation set, for which the AAD takes values around 4%: docosane, octacosane, and triacontane. These can be considered adequate results, as the MAXD are always below 9.3%. As shown by Mulero et al.,^36^ in the case of docosane, the percentage deviations are high at temperatures around 550 K because the data provided by DIPPR^37^ and DETHERM^38^ databases do not agree well, although they follow a similar trend. Similarly, for octacosane, there is a certain disagreement between the data given by DIPPR^37^ and by Koller et al.^95^ (obtained using the surface light scattering experimental method), as they do not follow the same trend.^36^ This leads to a MAXD value of 8.86% when the EoS model is applied, as shown in Table S2. The same occurs for triacontane, but in this case, the disagreement occurs^36^ between the DIPPR data and those obtained experimentally by Klein et al.^16^ by using a surface light scattering method (see details at ref (36)). As shown in Figure 3, the model tries to connect the data at low temperatures, where two different but similar trends are observed, and those at high temperatures, where only DIPPR data are available.^36^ Despite the differences observed between the model and the data, the PDs are below 9%, as seen in Figure 4.

In sum, the MAXD values around 9% obtained for these three fluids are not due to the bad behavior of the model but due to certain disagreements between the data obtained from different sources.

**Figure 5:**
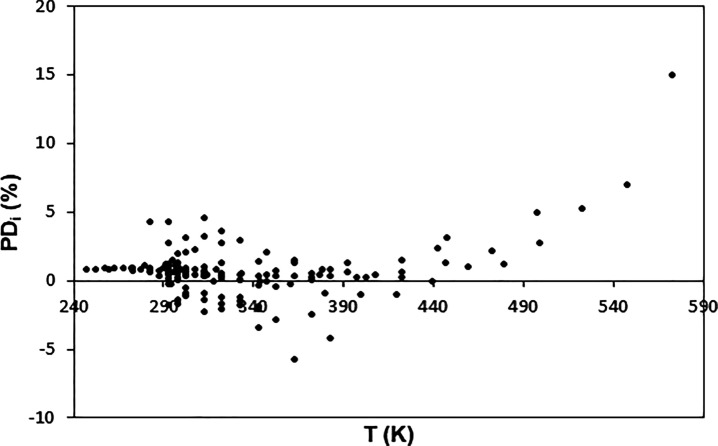
Percentage deviations between the values for the surface tension of decane obtained with the model and those selected in the database.

**Figure 6:**
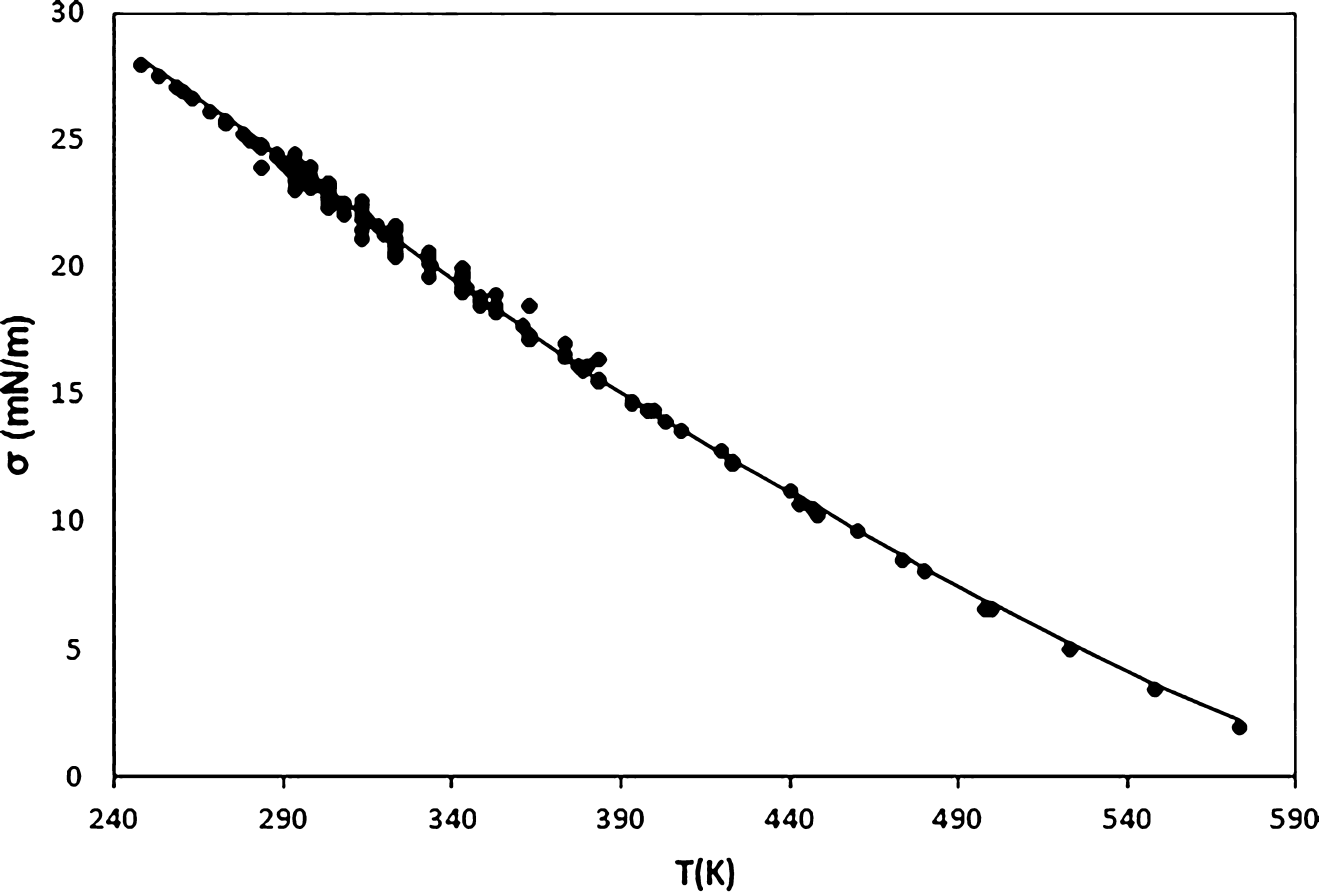
Surface tension values for decane vs the temperature. Points: selected data. Line: results for the model.

There are three *n*-alkanes for which the MAXD values are higher than 10%: decane, eicosane, and hexacosane. As shown in Figure 5, in the case of decane, only for a datum out of the 149, the PD_i_ is higher than 10%. This maximum corresponds to the highest temperature considered. As Mulero et al.^36^ explained, for this fluid, the only surface tension data available at elevated temperatures was recently measured by Klein et al.^16^ using a surface light scattering method. They generally follow a similar trend to the data available at lower temperatures. Still, indeed these new data have not been compared with others, and it has been observed that for some fluids, there are apparent disagreements between the data obtained by scattering and the values obtained for other methods.^36^ This means that the results at high temperatures must be analyzed with caution. In any case, the model proposed here gives an AAD of only 1.27% for this fluid and can be considered as very accurate except perhaps at the highest temperatures considered. The general agreement between the model and the selected data can be observed in Figure 6.

In the case of eicosane, 25 data were selected in the temperature range from 313.15 to 618.12 K. Nevertheless, they are not homogeneously distributed in temperatures,^36^ as 24 of them are in the range from 313.15 to 393.16 K (at atmospheric pressure), and there is a datum located at 618.12 K (at a saturation pressure slightly higher than the atmospheric one), from Lielmezs and Herrick.^96^ Consequently, the percentage deviations are below 3.6% for the first 24 data but 15.15% for the latest one. That means that the proposed model cannot adequately connect the data at the low temperatures with the datum at the highest temperature, which cannot be compared with other sources. In any case, the AAD is 2.27%, and the model can be considered very appropriate, except for the highest temperature considered.

Finally, in the case of hexacosane, the data at low and high temperatures come from different sources.^36^ This can explain that the PD_i_ are very low at low temperatures and higher than 10% only for the datum at the highest temperature (13.24% at 691.78 K). In any case, the AAD is as low as 1.53%, which is an excellent result for a model containing only general parameters (not specific for each fluid).

In sum, the model gives deviations higher than 10% for the correlation set of fluids only for 5 data out of 1116 considered. These high percentage deviations are obtained at high temperatures, as was previously explained. The surface tension takes values near zero, and then any tiny absolute deviation can lead to a high percentage deviation. Previous paragraphs have explained and justified the possible origin of these and other high deviations. The obtained AADs and overall AAD (2.43%) can be considered adequate results compared with the obtained using other general methods summarized in Table S1.

### 4.2 Pure *n*-Alkane Results for the Testing Set

The testing set includes 13 *n*-alkanes (listed in Tables S3 and 5), which were not included in the calculation of the model parameters, and for which a total of 1313 data at Tr below 0.93 were selected. The number of data for each fluid goes from 9 to 339, but there is no relationship between the number of data and the obtained relative deviations. When the EoS model is applied to predict the selected values, the overall AAD is 2.30%, that is, it is of the same order as that obtained for the correlation set and other kinds of models (see Table S1).

The AADs are below 3.3% except for hexatriacontane (7.37% in this case). Moreover, the MAXDs are below 9.3% except for hexane, heptane, and hexatriacontane. Comparing the results obtained with other models included in Table S1 can be considered excellent as the surface tension values are fully predicted.

In the case of hexatriacontane, there are only nine data available,^36^ coming from DIPPR and obtained by using Sugden’s method. This means that the comparison is made between two sets of data (the DIPPR one and the obtained with the proposed EoS model) that are entirely predictive. An AAD of 7.37% means that the two methods lead to slightly different values in most of the temperature range selected. As the AADs for the rest of the fluids are clearly lower, this means that for hexatriacontane, one of the two used models (Sugden or EoS one) does not follow the same trend for the rest of the rest *n*-alkanes. New experimental data are necessary to clarify what model is in better agreement.

**Figure 7:**
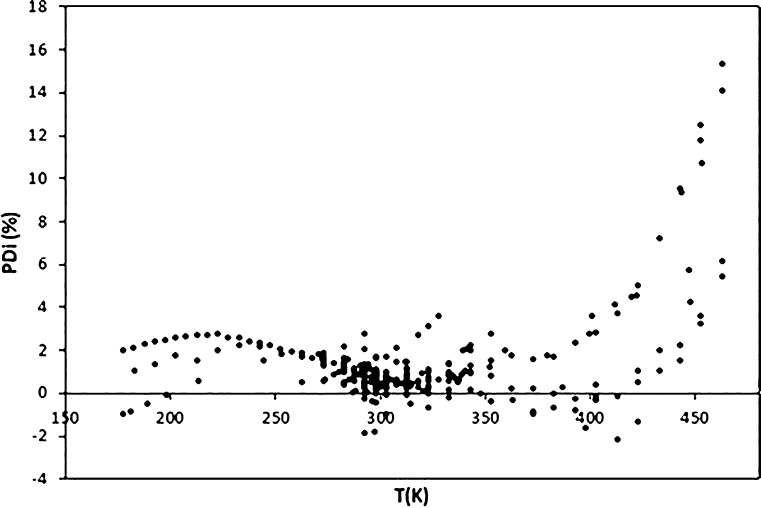
Percentage deviations between the values for the surface tension of hexane obtained with the model and those selected in the database.

**Figure 8:**
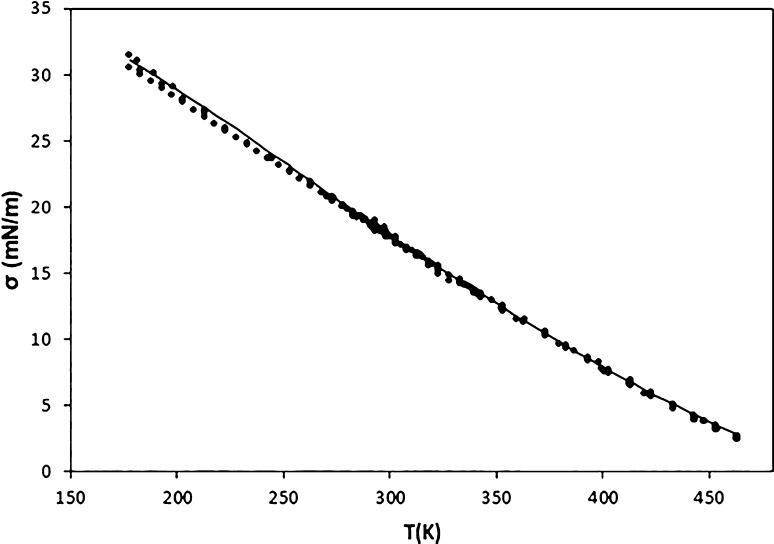
Surface tension values for hexane versus the temperature. Points: selected data. Line: results for the model.

In the case of hexane, a total of 259 data were selected^36^ by using more than 28 sources. As can be seen in Figure 7, the highest PDs are found at high temperatures, where different authors provide slightly different surface tension values. Thus, PDs higher than 10% are found for 5 data in the temperature range from 453.15 to 463.28 K. Nevertheless, there are other data for which lower deviations are obtained in this same temperature range. Despite this, the AAD is only 1.53% due to the excellent behavior of the model in the rest of the temperature range, as shown in Figure 8.

A high MAXD, 12.59%, is also obtained for heptane, for which 339 data were selected from multiple sources. This is the only PD_*i*_ higher than 10% for the temperature range considered, and it must be noted that lower PDs are obtained for similar temperatures. This means that a datum at 497.06 K disagrees with other ones at similar temperatures compiled from different sources. As shown in Table 3, the AAD is just 1.27%, which is a very low value when considering the number of data and the extension of the temperature range considered.

In sum, if the 32 *n*-alkanes are considered as one only set, the overall AAD obtained by using the proposed model is 2.38%, which is a very low value if one takes into account the number of data selected, the different origins of these data, the temperature range considered, and the fact that predictions are made for 13 *n*-alkanes (1313 data). The comparison with other models mentioned in the introduction and Table S1 is also very favorable. PDs higher than 10% are obtained only for a few data, and the cause of these deviations can be explained by their origin (source used to obtain them). As shown in Table S1, some previous models give AADs higher than 10% for some *n*-alkanes. In comparison, for the proposed EoS model, the highest value is 7.37% (for hexatriacontane). The cause of this deviation can be explained by considering that the comparison is made between values predicted by two different methods. Moreover, it must be taken into account that most previous models do not include prediction or testing for other substances different from those used to calculate the adjustable parameters. Finally, these results show that the EoS model proposed by Cardona and Valderrama^88^ can be extended to temperatures higher than *T*_b_ by using the values of the saturation pressure as inputs.

### 4.3 Pure *n*-Alkane Results at the Highest Temperatures

As previously mentioned, the model defined in Table 1 has been applied only for reduced temperatures below 0.93. At higher temperatures, the geometric similitude is not observed (Figure 1), and the surface tension goes to zero (and it is zero at *T*_c_).

To show that the proposed model is physically correct, that is, it gives surface tension values very near to zero at temperatures near the critical point, the mean and maximum absolute differences between the calculated values and the selected data have been calculated and are listed in Table 4. As can be seen, only for the first eight *n*-alkanes there are available^36^ data at *T*_r_ > 0.93.

**Table 4:** Mean Absolute Difference between the Calculated Values and the Selected Data at *T*_r_ > 0.93^a^

substance	|σ(*T*_*i*_) – σ_*i*_|/*N*^′^ (N m^–1^)	max{|σ(*T*_*i*_) – σ_*i*_|} (N m^–1^)
methane	2.82 × 10^–5^	8.90 × 10^–5^
ethane	4.05 × 10^–5^	1.11 × 10^–4^
butane	1.48 × 10^–4^	2.20 × 10^–4^
pentane	1.07 × 10^–4^	2.08 × 10^–4^
propane	7.67 × 10^–5^	2.01 × 10^–4^
hexane	1.78 × 10^–4^	3.61 × 10^–4^
heptane	1.45 × 10^–4^	2.99 × 10^–4^
octane	2.50 × 10^–5^	3.08 × 10^–5^

a *N*′ is the number of data selected in that temperature range.

As shown in Table 4, the mean and maximum absolute differences are of the order of 10^–4^ or 10^–5^ N m^–1^. In particular, the highest maximum absolute difference is 3.61 × 10^–4^ N m^–1^ and the highest mean difference is 1.78 × 10^–4^ N m^–1^, obtained in both cases for hexane. This means that the model gives adequate values (going properly to zero as the temperature increases) for the surface tension when extrapolated at reduced temperatures higher than 0.93. In any case, the percentage deviations in this temperature range can be high because the surface tension values are very near to zero.

### 4.4 Results for Mixtures

The EoS model can be applied to binary and ternary mixtures by including the mixing rules shown in row (AA4) of Table 1. No adjustable interaction parameters are used, so the results obtained are entirely predictive. They are based exclusively on the model developed for some pure fluids (only 19 out of the 32 *n*-alkanes considered here).

Results for 11 binary mixtures and 4 ternary mixtures are shown in Table 5. For each mixture, the data were taken from only one source,^47,48,62,64,79,97,98^ and they were measured at atmospheric pressure. At least 20 data have been selected for each mixture, whereas the maximum data number is 45 for binary mixtures and 60 for ternary ones. In most cases, the concentration range includes the pure fluids, that is, Δ*x*_1_ goes from 0 to 1.

**Table 5:** Data and Results for Mixtures^a^

mixtures	*N*	Δ*T* (K)	Δσ_m_ (mN m^–1^)	Δ*x*_1_	Δ*x*_2_	AAD (%)	MAXD (%) [*T*_m_ (K) – *x*_1m_ – *x*_2m_ – *x*_3m_]	refs
hexadecane + eicosane	31	303.15–343.15	23.51–27.63	0–1		3.82	9.03 ([343.15] – 1)	(79)
hexane + decane	42	303.15–353.15	12.09–22.87	0–1		1.14	3.79 ([353.15] – 1)	(97)
pentane + hexadecane	45	293.15–323.15	11.95–19.62	0.17–1		3.67	8.84 ([323.15] – 1)	(47)
pentane + heptane	45	293.15–323.15	11.95–19.62	0.17–1		3.67	8.84 ([323.15] – 1)	(47)
eicosane + decane	34	293.15–343.15	19.66–27.58	0–1		2.17	4.35 ([343.15] – 0.4)	(64)
docosane + decane	26	313.15–343.15	19.66–27.42	0–1		2.99	5.7 ([313.15] – 0.2)	(64)
tetracosane + decane	22	313.15–343.15	19.66–27.14	0–1		4.04	6.98 ([343.15] – 0.4)	(64)
heptane + hexadecane	25	293.15–333.15	16.5–28.12	0–1		2.90	5.5 ([323.15] – 0.5)	(79)
heptane + eicosane	20	313.15–343.15	15.32–27.58	0–1		5.98	12.2 ([343.15] – 0.75)	(62)
heptane + decane	25	293.15–333.15	16.5–24.47	0–1		0.80	1.88 ([293.15] – 0)	(79)
decane + hexadecane	25	293.15–333.15	20.6–28.12	0–1		1.64	5.12 ([293.15] – 0)	(79)
decane + eicosane + tetracosane	22	313.15–343.15	21.02–27.27	0–0.8	0.1–0.5	4.49	6.77 ([343.15] – 0.6 – 0.2 – 0.2)	(64)
heptane + eicosane + tetracosane	25	313.15–343.15	18.52–27.27	0–0.8	0.1–0.5	10.43	14.05 ([333.15] – 0.602 – 0.199 – 0.199)	(62)
hexane + decane + hexadecane	24	303.16	19.35–24.79	0–0.6	0.07–0.949	8.44	22.12 ([303.16] – 0.6002 – 0.0698 – 0.33)	(98)
hexadecane + heptane + pentane	60	293.15–323.15	11.96–27.57	0–1	0–1	8.51	15.33 ([323.15] – 0.42 – 0.248 – 0.332)	(48)

a *N* is the number of data obtained from the references given in the last column. The ranges of temperature, surface tension, and composition are given as Δ*T* (K), Δσ_m_ (N m^–1^), Δ*x*_1_, and Δ*x*_2_ (this last only for ternary mixtures). AAD and MAXD have the same meaning as in Table 3. [*T*_m_ (K) – *x*_1m_ – *x*_2m_ – *x*_3m_] indicate the location of the maximum deviation.

Table 5 shows the AADs model’s predictions when applied to binary mixtures. It is remarkable how these AADs are of the same order of magnitude as those calculated for the pure fluids, even though the model is fully predictive in this case. The overall AAD for binary mixtures (340 data) is 2.98%, and the AADs range from 0.80% to 5.98%, with values below 3% for 6 out of the 11 mixtures considered in this study. These AAD values are in the same order as those given by other predictive models listed in Table S2. Moreover, the MAXDs are below 10% for 10 binary mixtures, which can be considered an excellent prediction result.

The lowest AAD and MAXD values are obtained for heptane + decane (0.80 and 1.88%, respectively), for which 25 data were considered, and for hexane + decane (1.14 and 3.79%, respectively), for which 42 data were calculated. The temperature ranges for the data of these two mixtures are like those considered for other binary mixtures, as included in Table 5.

**Figure 9:**
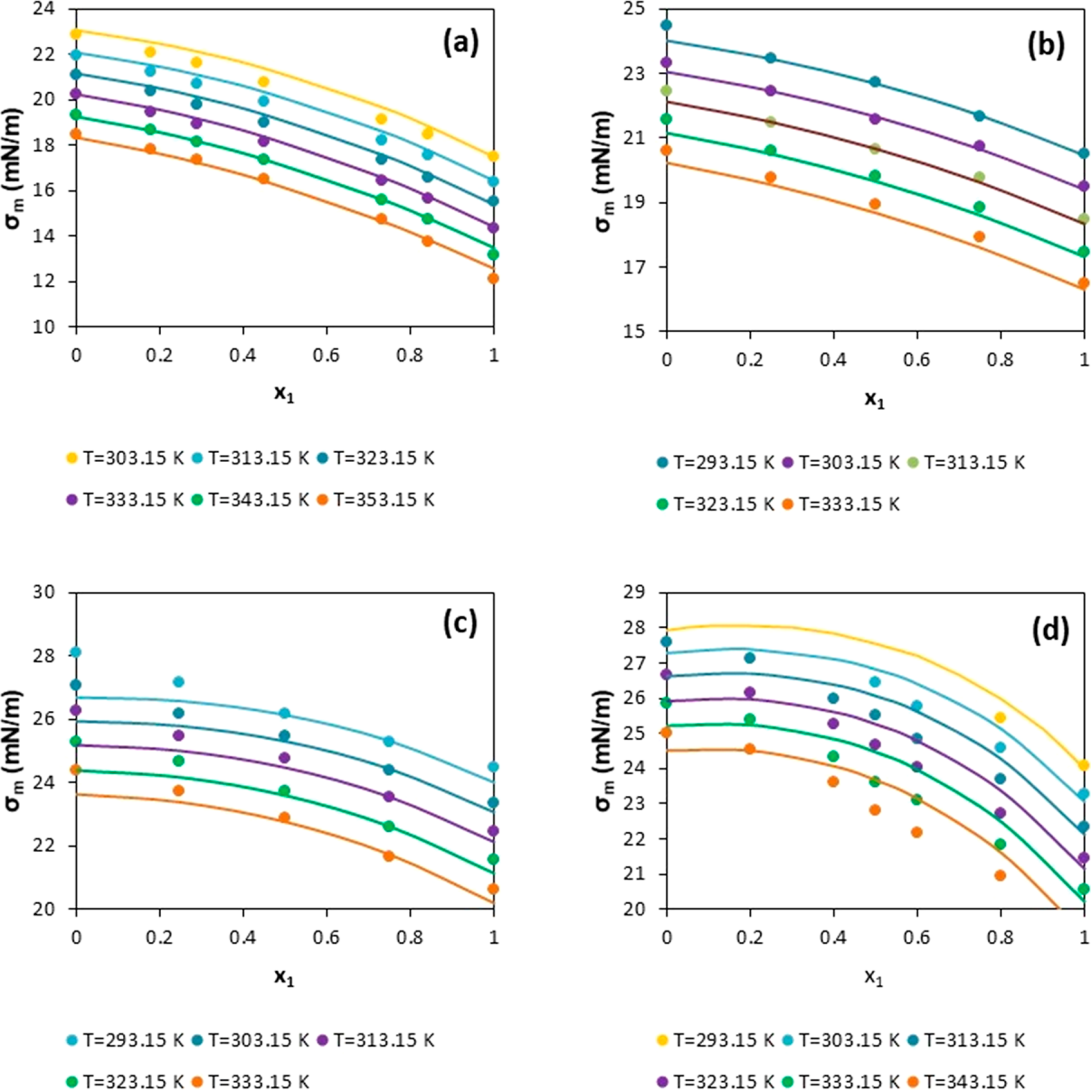
Surface tension values for different types of mixtures versus molar fraction for different temperatures. (a) Hexane + decane; (b) heptane + decane; (c) decane + hexadecane; and (d) eicosane + decane. Points: selected data (details in Table 5). Lines: prediction results from the proposed model.

The behavior of the predictive model for four mixtures containing decane (including the two mentioned in the previous paragraph) is illustrated in Figure 9. As can be seen for some mixtures, the highest deviations are obtained when the corresponding pure fluids are considered, that is, for *x*_1_ = 0 or = 1 (see details in Table 5). As an example, it is clear that in the mixture containing hexadecane, the source of disagreement at 293.15 K is the corresponding value reported by Rolo et al. for the pure component.^79^ This value is higher than the other 10^36^ considered in the pure EoS model. In any case, the percentage deviation at this temperature is just 5.12%, which can be regarded as a good predictive result.

A similar situation occurs for the mixture eicosane + decane (Figure 9d). The model tries to reproduce the average value used in the determination of the pure component, which is lower than the corresponding one reported for the mixture. Consequently, this leads to a high curvature in the lines representing the model’s behavior. Here, the MAXD is at *x*_1_ = 0.4 but it is only 4.35% (see Table 5).

On the other hand, the highest deviations between the selected values and the model predictions are observed for heptane + eicosane and tetracosane + decane mixtures.

In particular, the highest AAD for binary mixtures, 5.98%, is found for heptane + eicosane. In this mixture, PDs higher than 10% are obtained for 7 out of the 20 data selected, and the MAXD is 12.20%. In all cases, these higher deviations are located at *x*_1_ values around 0.75 and 0.5, but low deviations are obtained for the pure fluids, that is, when *x*_1_ is 1 or 0. In any case, this is the only binary mixture for which the model gives MAXD values higher than 10%.

**Figure 10:**
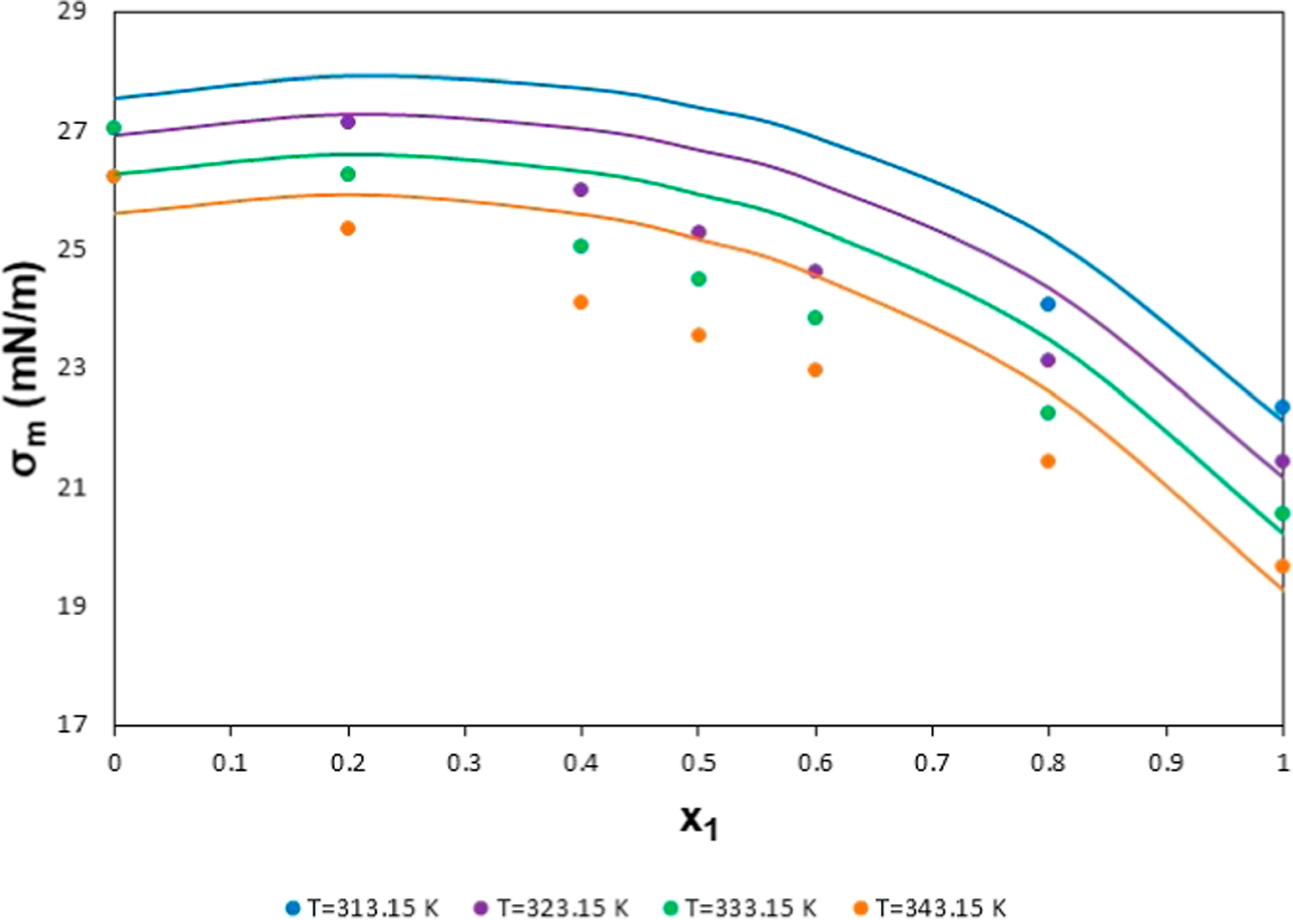
Surface tension values for the mixture tetracosane + decane vs concentration (mol fraction of tetracosane) at several temperatures. Points: selected data (details in Table 5). Lines: results for the model.

The model’s behavior for all the data selected for tetracosane + decane is shown in Figure 10. The lines representing the model have an excessively high curvature and can reach a maximum at low-fraction molar values, which does not agree with the behavior of experimental data. As explained before, this is due to the lower surface tension value predicted for the pure fluid, which considers more data sources than the corresponding one given in the mixture reference data. Thus, the model tries to reproduce the mean selected values at *x*_1_ = 0 and = 1 and not just the values obtained in the sources mentioned in Table 5. This leads to the odd behavior of the predictive model at intermediate fraction molar values. Although the deviations between the lines and points in Figure 10 can be seen as high, as shown in Table 5, the MAXD for tetracosane + decane is slightly below 7%, which can be considered as a good predictive result.

The results for the mixtures with the highest AAD can be improved by introducing one adjustable parameter *k*_*ij*_ in the calculation of *a*_*m*_ in eq A4 of Table 1. This is, by replacing *a*_*ij*_ by *a*_*ij*_(1 – *k*_*ij*_). Thus, for instance, in the tetracosane + decane mixture the excessive curvature of the lines representing the model (shown in Figure 10) disappears by using *k*_12_ = −0.353. Then the AAD decreases from 4.04 to 0.94%, whereas the MAXD is just 2.88% instead of 6.98%. In any case, the interest here is to study the predictive behavior of the model, so no further fits were made.

In sum, for binary mixtures, the predictions agree with the model with maximum deviations below 12.3% despite the disagreement observed for some mixtures between the value of the surface tension for pure fluids given in the sources selected for mixtures and the corresponding mean value considered from different sources when the pure fluids are studied separately.

As expected from a general model including simple mixing rules,^100^ higher deviations are found in the case of the ternary mixtures.

The mixture hexane + decane + hexadecane (see Table 5) shows a MAXD of 22%, but only 9 out of the 24 data selected have PDs higher than 10%. These deviations are comparable with the obtained by Pandey and Pant^98^ by using Flory’s statistical theory (with MAXD of 15.30%) and by Mishra and Tripathi^99^ using a corresponding-states relationship (with MAXD of 12.30%) for the same mixture. With the proposed model, the AAD is 8.44%, which can be considered an acceptable value for predicting the surface tension of ternary mixtures.

As shown in Table 5, the highest AAD value for ternary mixtures is found for heptane + eicosane + tetracosane. This is an expected result, provided that the highest AAD value for binary mixtures was obtained for heptane + eicosane (see Table 5 and paragraphs above).

In the case of hexadecane + heptane + pentane, PDs higher than 10% are obtained for 23 of 60 data selected, with 15.29% MAXD. In this case, the final AAD is 8.51%, that is, similar to the obtained for hexane + decane + hexadecane. It can be considered a good predictive result considering the high number of data.

Finally, the best result for ternary mixtures is found for decane + eicosane + tetracosane, being the deviations of the same order to those obtained for binary mixtures.

Obviously, the previous results can be improved by introducing adjustable interaction parameters in eq A4 of Table 1. Three adjustable parameters are needed for ternary mixtures, and two possible strategies to obtain them are possible. One procedure is to use the *k*_*ij*_ values obtained for binary mixtures, when available, and then apply the model for predictions of ternary mixture results. In this case, the obtained AADs for ternary mixtures are similar to those found for binary ones. The second strategy could be to use adjustable values for *k*_*ij*_ obtained directly from the data selected for the ternary mixtures. In this case, the AADs are even lower and, in fact, are of the same order as those given by other more specific models listed in Table S2.

## 5 Conclusions

By means of the geometric similitude concept, a pressure–surface tension–temperature EoS is proposed for *n*-alkanes. This model includes adjustable parameters obtained using the selected data for 19 *n*-alkanes randomly chosen. The model can predict the surface tension values for the other 13 *n*-alkanes not considered in the parameter fitting. The overall AAD is 2.4% for the whole fluid set (32 *n*-alkanes and 2429 surface tension data) at temperatures below 0.93 times the critical point. Despite the limitation on temperature, it is the first model of this kind that can be applied to pressures above the atmospheric one. Moreover, it has been pointed out that at temperatures higher than 0.93 *T*_c_, the model gives surface tension values very near to zero.

It has been shown that the model for pure fluids can be easily extended to mixtures by considering simple mixing rules, which do not include new parameters. In particular, surface tension values for 11 binary mixtures have been predicted with an overall AAD of 2.98%. Only 7 out of the 340 data selected for the binary mixtures have percentage deviations slightly higher than 10%, which is an excellent result for a predictive model. Moreover, 131 data for four ternary mixtures have been predicted with an overall AAD of 7.97%.

In general, the model fulfils the proposed requirements because it is based on a complete, consistent, and updated selection of data for pure fluids, it can be applied in a wide range of temperatures and pressures, it can be used for predictions, and it can be easily extended to mixtures providing good overall results.

## Supporting Information Available

Information about the selected empirical or semiempirical general models proposed in the literature for estimating the surface tension of pure organic compounds and their mixtures and values from DIPPR^37^ for the molar mass and critical properties used as inputs in the model for every *n*-alkane (PDF)
