# Supplementary material for: Pressure–Surface Tension–Temperature Equation of State for n-Alkanes
Source: Ind Eng Chem Res. 2022 Feb 23;61(9):3457–73. doi: 10.1021/acs.iecr.1c04979 (PMC8919510; doi:10.1021/acs.iecr.1c04979)
Supplement: Supplementary file 1 — ie1c04979_si_001.pdf [file ie1c04979_si_001.pdf]

A pressure-surface tension-temperature equation of state for *n*-alkanes

A. Mulero[1,*], I. Cachadiña Gutiérrez[1]

[1]Departamento de Física Aplicada, Universidad de Extremadura, 06006 Badajoz, Spain. https://ror.org/0174shg90 (mulero@unex.es and icacha@unex.es)

L.F. Cardona[2], J. O. Valderrama[3]

[2]Universidad Católica Luis Amigó, Departamento de Ciencias Básicas, Transversal 51A No. 67B-90, Medellín, Colombia (luis.cardonapa@amigo.edu.co)
[3]Center for Technological Information (CIT), Monseñor Subercaseaux 667, La Serena-Chile (joseovalderrama@gmail.com)

SUPPORTING INFORMATION

Table S1: Selected empirical or semi-empirical general models proposed in the literature for estimating the surface tension of organic compounds. N_{data} = number of data used. Range of temperatures, including all the fluids. Sources are given only if they are one or two; in other cases, multiple sources are used. . N_{par} = Number of adjustable parameters for the model or each fluid. Deviation AAD is defined in Eq. (1) for a substance. The overall AAD is the mean value for all the substances. AADs are the range of AAD obtained for the fluids. MAXD is the maximum percentage deviation for the data.

**Year[Ref.]**	**Model**	**Number and type of Substances**	N_{data} ***(Approx. Range of T) (Source of data)***	N_{par} **(model or fluid)**	**Deviations**
1997[57]	Corresponding-states model with two reference fluids (methane and octane)	86 hydrocarbons: alkanes, alkenes, branched alkanes, aromatics, cycloalkanes	1051 (68 K - 523 K)	4 (model)	Overall AAD = 2.74% AADs for *n*-alkanes: 0.5%-11.4%
1998[58]	Corresponding-states model	41 hydrocarbons: 16 *n*-alkanes, 16 1-alkenes, 4 cycloalkanes, 5 aromatics	490 (260 for *n*-alkanes)	4 (model GSTE4 for *n*-alkanes)	Overall deviation for *n*-alkanes = 0.14 mN/m
2000[59]	Corresponding-states model	9 substances: nitrogen, first 8 *n*-alkanes, and *i*-butane	694 (90 K - 540 K)	4 (model)	Overall AAD = 3.7%
2001[61]	Corresponding-states model with 3 reference fluids (*n*-hexane, *n*-undecane and *n*-pentadecane)	19 *n*-alkanes	275 (129 K - 501 K)	6 (model)	Overall AAD = 1.14% AADs = 0.21%-5.48%

2001[61]	Extended Corresponding-states model with shape factors and with different reference fluids (*n*-pentane,<br>*n*-decane, or *n*-pentadecane) for each target fluid	18<br>*n*-alkanes	271<br>(129 K - 501 K)	5 (model)	Overall AAD = 3.7%<br>AADs = 0.3% - 13%
2013[69]	Two corresponding states models	Around 1700 substances. (Number of *n*-alkanes not indicated)	495 for *n*-alkanes (DIPPR database, temperature range not indicated)	1 (model 1) and 2 (model 2)	Overall AADs for *n*-alkanes = 4.9% and 2.7%
2015[72]	Surface tension model based on vaporization enthalpy and sphericity factor	25 *n*-alkanes	472<br>(86.15 K to 673.15 K)<br><br>(NIST Webbook model)	2 (model)	Overall AAD = 4.61%.<br>AADs = 1.18%-8.66%
2016[73]	A group contribution model based on the Guggenheim-Katayama-van der Waals equation	50 hydrocarbons:<br>linear alkanes, alkyl alkanes, alkyl cycloalkanes	394<br>(273.15 K to 333.15 K, TRC database by NIST, 2008)	18 (model)	(Just for eight fluids and 2 data for each fluid)<br>Overall AAD = 1.57%.<br>MAXD = 3.3%
2016[74]	Lielmezs- Herrick corresponding-states model	28 hydrocarbons:<br>9 *n*-alkanes, and aromatic hydrocarbons, cycloalkanes, alkenes	Not reported (REFPROP program)	2 (model)	Overall AAD = 2.40%.<br>AADs for *n*-alkanes = 2.11% - 6.87%

2016[74]	Lielmezs- Herrick correlation	28 hydrocarbons:<br>9 *n*-alkanes, and aromatic hydrocarbons, cycloalkanes, alkenes	Not reported (REFPROP program)	2 (each fluid)	Overall AAD = 1.69%.<br>AADs for *n*-alkanes = 0.73% - 7.20%
2016[75]	Parachor model with an EoS and a group contribution method	40 hydrocarbons:<br>*n*-alkanes, alkenes, branched alkanes, cycloalkanes, cycloalkenes, benzene, and its alkyl derivatives	350<br>(NIST and Dortmund DDBST databases)	7 (model)	Overall AAD (all fluids) = 6.54%<br>Overall AAD for 11 *n*-alkanes used in correlation = 10.84%<br>AADs for 11 *n*-alkanes = 4.56%-26.35%
2017[76]	General correlation with the viscosity	21 *n*-alkanes	418<br>(173.15 K to 673.15 K) (NIST Webbook model)	3 (model)	Overall AAD = 5.66%.<br>AADs = 2.34% - 14.27%
2018[77]	Artificial neural network model	91 hydrocarbons<br>37 *n*-alkanes, 37 1-alkenes, 17 cycloalkanes	5461<br>(134.86 K to 1068.24 K for *n*-alkanes)<br>(Yaws and Gabbula correlation[78] for each fluid)	136 (ANN model)	Deviations for *n*-alkanes = -0.59 mN/m to 0.51 mN/m.<br>(not percentage values are given)

Table S2: Selected empirical or semi-empirical general models applied to the surface tension of mixtures of organic compounds. N_{par} is the number of adjustable parameters used for each model or mixture without considering the parameters used for pure fluids.

Year[Ref.]	Model	Number and type of mixtures	n_{data} *(Range of T)*	N_{par}	Deviations
1997[57]	Corresponding-states model with two reference fluids (methane and octane)	24 binary and three ternary and two ten-component mixtures composed of hydrocarbons, halogenated and noble gases.	603 (83.82 K-353 K)	0 (prediction)	Overall AAD = 1.77% AADs (mixtures of *n*-alkanes) = 0.77-3.21%
2002[79]	Corresponding-states model with three reference fluids (heptane, decane, and hexadecane)	Four binary mixtures composed of heptane, decane, hexadecane, and/or eicosane.	106 (293.15 K-343.15 K)	0 (prediction)	Overall AAD = 0.97% AADs (mixtures of *n*-alkanes) = 0.44-2.06%
2003[62]	Corresponding-states model with 3 reference fluids (heptane, decane, and eicosane)	18 binary and 2 ternary mixtures composed of *n*-alkanes	(not available)	1 (model)	Overall AAD = 1.53% for symmetric mixtures, 1.08% for asymmetric ones. AADs = 0.48-4.48%
2004[63]	Pelofsky empirical relation between surface tension and viscosity	6 binary and 2 ternary mixtures containing decane and heavier *n*-alkanes.	20 (293.15 K-343.15 K)	2 (model)	Overall AAD = 3.20% AADs = 1.2%-8.5% MAXD = 13.2%

2005[64]	Corresponding-states model with 3 reference fluids (heptane, decane, and eicosane)	Three binary and one ternary mixture containing decane, eicosane, docosane, and/or tetracosane	81 (293.15 K-343.15 K)	0 (prediction)	Overall AAD = 1.20% AADs = 0.7%-2.0%
2013[80]	Sprow–Prausnitz equation	154 binary mixtures including hydrocarbons of different kinds (3 mixtures of *n*-alkanes).	2058 (298 K, except for heptane+eicosane at 323 K and heptane+hexadecane at 293 K)	0 or 2 (each mixture)	Overall AAD = 1.55% AADs (*n*-alkanes mixtures) = 0.04 %-7.57 %. MAXD = 14.43 %.

Table S3. Values of the molecular mass molecular mass, critical pressure, critical temperature, and critical compressibility factor for every *n*-alkane. Values are taken from DIPPR.[37] The substances are sorted by the number of carbons.

	Substance	CAS#	molar mass and critical properties			
			M (g/mol)	Tc (K)	Pc (bar)	Zc
Correlation	methane	74-82-8	16.043	190.56	45.99	0.286
	ethane	74-84-0	30.069	305.32	48.72	0.279
	butane	106-97-8	58.122	425.12	37.96	0.274
	pentane	109-66-0	72.149	469.7	33.7	0.27
	nonane	111-84-2	128.255	594.6	22.9	0.255
	decane	124-18-5	142.282	617.7	21.1	0.254
	undecane	1120-21-4	156.308	639	19.5	0.252
	tridecane	629-50-5	184.361	675	16.8	0.247
	tetradecane	629-59-4	198.388	693	15.7	0.244
	pentadecane	629-62-9	212.415	708	14.8	0.244
	eicosane	112-95-8	282.547	768	11.6	0.243
	heneicosane	629-94-7	296.574	778	11.1	0.242
	docosane	629-97-0	310.601	787	10.6	0.24
	tetracosane	646-31-1	338.654	804	9.8	0.239
	hexacosane	630-01-3	366.707	819	9.1	0.238
	heptacosane	593-49-7	380.734	826	8.83	0.239
	octacosane	630-02-4	394.76	832	8.5	0.237
	triacontane	638-68-6	422.813	844	8	0.237
	dotriacontane	544-85-4	450.866	855	7.5	0.236

**Testing**	propane	74-98-6	44.096	369.83	42.48	0.276
	hexane	110-54-3	86.175	507.6	30.25	0.266
	heptane	142-82-5	100.202	540.2	27.4	0.261
	octane	111-65-9	114.229	568.7	24.9	0.256
	dodecane	112-40-3	170.335	658	18.2	0.251
	hexadecane	544-76-3	226.441	723	14	0.243
	heptadecane	629-78-7	240.468	736	13.4	0.244
	octadecane	593-45-3	254.494	747	12.7	0.243
	nonadecane	629-92-5	268.521	758	12.1	0.242
	tricosane	638-67-5	324.627	796	10.2	0.24
	pentacosane	629-99-2	352.68	812	9.5	0.24
	nonacosane	630-03-5	408.787	838	8.26	0.238
	hexatriacontane	630-06-8	506.973	874	6.8	0.237
